# The Importance of Therapeutic Time Window in the Treatment of Traumatic Brain Injury

**DOI:** 10.3389/fnins.2019.00007

**Published:** 2019-01-23

**Authors:** Maliheh Mohamadpour, Kristen Whitney, Peter J. Bergold

**Affiliations:** ^1^Department of Neurology, State University of New York Downstate Medical Center, Brooklyn, NY, United States; ^2^Department of Physiology and Pharmacology, State University of New York Downstate Medical Center, Brooklyn, NY, United States

**Keywords:** drug testing, TBI pathophysiology, time to first dose, TBI severity, clinical trials, pharmacokinetics

## Abstract

Traumatic brain injury (TBI) is a major cause of death and disability. Despite its importance in public health, there are presently no drugs to treat TBI. Many reasons underlie why drugs have failed clinical trials, one reason is that most drugs to treat TBI lose much of their efficacy before patients are first treated. This review discusses the importance of therapeutic time window; the time interval between TBI onset and the initiation of treatment. Therapeutic time window is complex, as brain injury is both acute and chronic, resulting in multiple drug targets that appear and disappear with differing kinetics. The speed and increasing complexity of TBI pathophysiology is a major reason why drugs lose efficacy as time to first dose increases. Recent Phase III clinical trials treated moderate to severe TBI patients within 4–8 h after injury, yet they turned away many potential patients who could not be treated within these time windows. Additionally, most head trauma is mild TBI. Unlike moderate to severe TBI, patients with mild TBI often delay treatment until their symptoms do not abate. Thus, drugs to treat moderate to severe TBI likely will need to retain high efficacy for up to 12 h after injury; drugs for mild TBI, however, will likely need even longer windows. Early pathological events following TBI progress with similar kinetics in humans and animal TBI models suggesting that preclinical testing of time windows assists the design of clinical trials. We reviewed preclinical studies of drugs first dosed later than 4 h after injury. This review showed that therapeutic time window can differ depending upon the animal TBI model and the outcome measure. We identify the few drugs (methamphetamine, melanocortin, minocycline plus N-acetylcysteine, and cycloserine) that demonstrated good therapeutic windows with multiple outcome measures. On the basis of their therapeutic window, these drugs appear to be excellent candidates for clinical trials. In addition to further testing of these drugs, we recommend that the assessment of therapeutic time window with multiple outcome measures becomes a standard component of preclinical drug testing.

## Therapeutic Time Window is a Key Element of Drugs to Treat TBI

Despite decades of research, there are currently no treatments for TBI other than palliative care (Diaz-Arrastia et al., 2014). The reasons for the lack of therapeutics are many; drug may have failed in clinical trials since most preclinical studies dose drugs immediately or soon after experimental TBI (Diaz-Arrastia et al., 2014). This experimental design fails to take into account the well-documented clinical phenomenon of treatment gap: the time individuals wait before seeking medical care after head trauma (Tanielian and Jaycox, 2008; Demakis and Rimland, 2010). In 1991, one quarter of an estimated 1.5 million patients in America did not seek medical care after receiving a TBI that did not result in death or long-term institutionalization (Sosin et al., 1996). The multiple reasons given to postpone or avoid treatment include perceived symptom resolution, as well as the time and cost of treatment (Demakis and Rimland, 2010). Military personnel are particularly at risk for TBI. Lack of access to safe and accessible transportation for deployed military personnel can delay treatment up to 72 h after TBI (Farmer et al., 2017). Thus, treatment gap likely contributes to negative outcomes after TBI. Despite the importance of treatment gap, we know relatively little about the time course of pathophysiological events that can be successfully targeted with drugs first dosed many hours to days following TBI.

The treatment of thromboembolic stroke using tissue plasminogen activator illustrates the importance of time window in neurodegenerative diseases with a rapid onset. Thromboembolic stroke produces a complex and rapidly evolving injury with an overlapping, yet distinct, pathophysiology to TBI. Tissue plasminogen activator (t-PA) is highly effective if dosed within 4.5 h of a stroke, yet its utility drops sharply after 4.5 h due to the increased probability of hemorrhage (Ahmed et al., 2013). Despite its established ability to prevent injury, only 2–5% of stroke patients receive t-PA (Miller et al., 2011). A major reason for the limited use of t-PA is its short time window (Miller et al., 2011). The experience of clinicians with t-PA to treat stoke suggests similar difficulties will arise if drugs to treat TBI have similarly short therapeutic time windows that fall off sharply.

Since no drug has received FDA-approval, a key unanswered question is: what is a clinically relevant therapeutic window for a TBI drug? Clinical trials at designated trauma centers have enrolled patients 4–7 h after a moderate to severe TBI. Even with the high skill of the clinical staff at these trauma centers; many patients could not be enrolled because treatment could not be initiated within 4–7 h. Less specialized hospitals are likely to have even longer treatment delays. To treat the largest number of patients, a drug or drug combination will likely need to retain high efficacy when first dosed 12 h after moderate to severe TBI. In contrast to those with severe or moderate TBI, patients with mild TBI often delay seeking medical help for days after injury until their symptoms do not abate (Sosin et al., 1996; Tanielian and Jaycox, 2008; Demakis and Rimland, 2010). Thus, drugs will need to retain efficacy when dosed days after injury to treat large numbers of patients with mild TBI.

Traumatic brain injury has a complex pathophysiology whether initiated by a blunt impact, penetration through the skull into the brain, or exposure to explosive blast (Dixon, 2017). TBI produces mechanical injury within seconds to neurons, glia, and vessels. This primary injury rapidly triggers a secondary injury that evolves for weeks to months (Dixon, 2017). Both primary and secondary injury damages both gray and white matter. Within minutes after primary injury, neurons lose the ability to control ion homeostasis, which results in accumulation of intracellular calcium, cell depolarization, excitotoxic release of glutamate and additional disruptions of ionic gradients (Weber, 2012; Guerriero et al., 2015). Impaired mitochondrial function leads to energy failure; calcium accumulation and elevated reactive oxygen species are additional early events in secondary injury (Bains and Hall, 2012; Weber, 2012; Hill et al., 2017). Damage to vessels reduces cerebral blood flow resulting in hypoxia, hypoglycemia, and breakdown of the blood-brain barrier (Price et al., 2016). Inflammation rapidly follows TBI and persists for weeks to months after injury (Hinson et al., 2015). Acute inflammation is initiated by release by necrotic cells of damage associated molecular patterns (DAMPS) that activate astrocytes and microglia. Release of proinflammatory cytokines and chemokines lead to further breakdown of the blood brain barrier and recruitment of peripheral inflammatory cells. Microglia and astrocyte activation occurs rapidly in both gray and white matter; neuroinflammation may become chronic and continue to injure brain for months or years after injury. Later events in secondary injury include induction of cytogenic and vasogenic edema, increased intracerebral pressure, oxidative damage and necrotic and apoptotic cell loss (Bains and Hall, 2012; Hill et al., 2017). Early events in white matter include damage to axons, impaired transport and swelling. Damage to oligodendrocytes leads to demyelination and oligodendrocyte loss (Narayana, 2017). White matter damage evolves for weeks resulting in Wallerian axonal degeneration.

The pathophysiological events of secondary injury are highly interconnected. If dosed before, or soon after TBI, a variety of drugs with diverse modes of action (anti-oxidants, glutamate receptor antagonists, and anti-inflammatories) greatly limit the scope of secondary injury (Diaz-Arrastia et al., 2014). These drugs are effective despite targeting only one component of secondary injury. This suggests that, early after TBI, multiple pathophysiological events trigger the spread of secondary injury. Thus, early blockade of any one of these many injury mechanisms results in a substantial, long-term therapeutic effect. As secondary injury evolves, the efficacy of most drugs rapidly diminish through loss of drug targets; the intensification of secondary injury greatly diminishes the therapeutic effect of inhibiting one injury mechanism. Drugs that retain efficacy when dosed at longer intervals after injury may target pathophysiological events that initiate slowly after injury. Alternatively, drugs with good therapeutic windows have multiple targets that can still reduce secondary injury even after its intensification over time.

The importance of therapeutic time window in treating TBI is illustrated by comparing the preclinical testing of progesterone and CDP-choline with the design of Phase III clinical trials testing the same drugs. Progesterone was tested in two recent Phase III trials for TBI. The PROTECT III trial (NCT00822900) recruited patients with moderate to severe TBI (Glasgow Coma Score 4–12) within 4 h post-injury while the SYNAPSE trial (NCT01143064) recruited patients with severe TBI (Glasgow Coma Score < 7) within 7 h (Skolnick et al., 2014; Wright et al., 2014). Both trials were unable to demonstrate a therapeutic effect for progesterone. Prior to Phase III testing, numerous laboratories demonstrated a diverse set of therapeutic effects of progesterone in multiple rodent TBI models (Stein and Sayeed, 2018). Progesterone reduced glutamate release, prevented vasogenic edema, restored the blood brain barrier, improved aerobic respiration, and increased myelin and neurotrophin synthesis. Most importantly, progesterone reduced brain damage and improved multiple functional outcomes. Few drugs have had such wide preclinical testing on so many therapeutic outcomes. Virtually all of these studies, however, first dosed progesterone within 1 h or less after injury (Stein and Sayeed, 2018). Only three studies dosed progesterone between 4 and 6 h after injury and none of these studies performed a careful analysis of how the efficacy of progesterone changed after injury (Peterson et al., 2012, 2015). A first dose of progesterone 4 h after experimental TBI decreased gray matter damage, improved motor function and limited astrocyte activation (Peterson et al., 2012, 2015). A first dose at 6 h produced small improvements on expression of Nogo-A, GFAP, and GAP-43 (Liu et al., 2014). None of these studies examined multiple therapeutic time windows so it remains unknown how the efficacy of progesterone changed with increasingly longer times to first dose. A study of a first dose of progesterone 1 or 6 h post-stroke showed good retention of drug efficacy in a rat cerebral ischemia model (Yousuf et al., 2014). Little is understood, however, of how the analysis of therapeutic time window in animal models of stroke tells us whether an equivalent therapeutic window exists for TBI. The PROTECT III and SYNAPSE trials provided important information of how rapidly we could recruit and treat patients after moderate to severe TBI, however, due to the lack of appropriate preclinical testing, we do not know if progesterone retained sufficient potency to treat TBI when first dosed at 4–7 h after injury.

The Phase 3 COBRIT study tested the efficacy of CDP-choline on mild, moderate and severe TBI (Zafonte et al., 2012). Most patients (86%) received drugs within the first 24 h after injury. The COBRIT study did not show improvement in any outcome measures. Compared to progesterone, there was relatively little preclinical testing of CHP-choline. Dixon et al. showed that a first dose of CDP-choline beginning 24 h after injury produced mild improvements on beam balance and beam walk, and on acquisition of Morris water maze (Dixon et al., 1997). Two additional studies that dosed CDP-choline immediately after injury reported decreased lesion volume, increased neuroprotection, improvements in neurological tests, edema and protection of the blood brain barrier (Başkaya et al., 2000; Dempsey and Raghavendra Rao, 2003). A potential hypothesized mechanism of action of CDP-choline was to improve lipid metabolism, yet no study examined whether CDP-choline limited white matter injury. As with progesterone, there are no studies examining the efficacy of CDP-choline at different therapeutic time windows. Thus, inadequate drug potency at the time when patients were treated may have contributed to the futility of the PROTECT III, SYNAPSE, and COBRIT trials.

## TBI Pathophysiology is a Major Determinant of Therapeutic Time Window

The speed of secondary injury after TBI results in the rapid appearance and disappearance of drug targets (Dixon, 2017). Studies of the therapeutic time windows of methyl-d-aspartate (NMDA) receptor agonists and antagonists illustrate how therapeutic time windows arise from the interaction of drugs with changes in TBI pathophysiological changes over time (Guerriero et al., 2015). Excessive glutamate release activates NMDA receptors within minutes after the onset of TBI (Guerriero et al., 2015). NMDA receptor activation produces calcium overload and activation of calcium-activated catabolic enzymes (Weber, 2012). If dosed soon after injury, NMDA antagonists prevent this calcium overload and prevent neuronal loss (Shohami and Biegon, 2013). The short therapeutic time window of NMDA receptor antagonists is the consequence of the speed of the calcium overload after TBI (Shohami and Biegon, 2013; Campos-Pires et al., 2015). Ongoing secondary injury subsequently produces a long-lasting downregulation of NMDA receptor expression. The loss of NMDA receptor function impairs synaptic plasticity and results in cognitive and memory deficits. The partial NMDA receptor agonist D-cycloserine when first dosed at 24 or 72 h post-injury improves Neurological Severity Score (NSS). A first dose of d-cycloserine at 24 h PI also improved performance of hippocampal-dependent tasks (Temple and Hamm, 1996; Adeleye et al., 2010; Sta Maria et al., 2017). A first dose of cycloserine at 24 h post-injury was also effective in rat model of pediatric TBI (Sta Maria et al., 2017). D-cycloserine improved performance on Novel Object Recognition and produced a mild improvement in acquisition, but not retention of Morris Water Maze (Sta Maria et al., 2017). Earlier dosing of d-cycloserine was ineffective at 8 or 16 h post-injury when NMDA receptors were downregulated (Adeleye et al., 2010). Thus, the different therapeutic time windows of NMDA receptor antagonists and agonists results from the differential consequences of NMDA receptor activation after TBI (Shohami and Biegon, 2013).

## Are Studies of Therapeutic Time Window in Animal Models Relevant to Human TBI?

Animal models of TBI have been invaluable for our understanding of TBI pathophysiology (Xiong et al., 2013). Most of the secondary injury events that occur in clinical TBI also occur in animal models. This has validated the use of animal models to find drug targets to treat TBI. Virtually all studies of therapeutic time window have used rodent TBI models (Table 1 and Supplementary Table 1). Studies of therapeutic time window in rodents not only assume similar TBI pathophysiology in animals and people, but that these pathophysiological events occur with similar kinetics. Both humans and rodents rapidly develop edema, elevated extracellular glutamate, excitotoxicity and elevated intracellular Ca^++2^ after TBI or experimental TBI (Palmer et al., 1993; Bullock et al., 1995; Vespa et al., 1998; Markgraf et al., 2001; Hutchinson, 2005; Weber, 2012). The increase in reactive oxygen species and its accompanying oxidative damage also occurs rapidly in animals and people (Bains et al., 2013; Cornelius et al., 2013). A variety of plasma biomarkers (GFAP, UCh-1, Tau, and S100β) show similar kinetics in rodent TBI models and clinical TBI (Mondello et al., 2016; Caprelli et al., 2017; Korley et al., 2018; Shahjouei et al., 2018). In both human TBI and TBI animal models, there is an acute and rapid increase in the levels of pro-inflammatory markers (Clausen et al., 2018; Huie et al., 2018). These data suggest that studies using rodent TBI model can provide important insights into the therapeutic window of a drug to treat clinical TBI.

**Table 1:** Drugs with a therapeutic time window of 12 h or greater in animal models of TBI.

Compound	TBI model	Species	Time windows tested post-injury	Longest first dose that improved outcomes	Mechanism of action	Reference
CDP-choline	CCI	Rat	24 h	Motor outcomes Navigation Decreased effect of muscarinic antagonists	Modulation of cholinergic neurotransmission, attenuation of lipid damage	Dixon et al., 1997
(RS)-2-chloro-5-hydroxyphenylglycine	CCI	Mouse	1 Month	Motor Outcomes Navigation LV White matter protection	mGluR5 agonist	Byrne et al., 2012
Cyclosporine A	CCI	Rat	0.25, 1, 6, and 24 h	LV 24 h	Protection of mitochondria	Sullivan et al., 2000
Cyclosporine A	CCI	Rat	1, 3, 4, 5, 6, and 8 h	LV 8 h	Protection of mitochondria	Sullivan et al., 2011
D-Cycloserine	WD	Mouse	8, 16, 24, and 72 h	NSS 72 h	NMADR partial-agonist	Adeleye et al., 2010
D-Cycloserine	FPI	Rat	24 h	Increased NMDA receptor expression NOR MWM	NMADR partial-agonist	Sta Maria et al., 2017
D-Cycloserine	FPI	Rat	24 h	MWM	NMADR partial-agonist	Temple and Hamm, 1996
FCCP	CCI	Rat	3, 6, and 24 h	Mitochondrial function 6 h Mitochondrial Ca^+2^ 24 h	Uncouples oxidative phosphorylation	Pandya et al., 2009
Fluasterone	CCI	Rat	0.5, 2, and 12 h	Motor function 12 h Navigation 12 h	Neurosteroid	Malik et al., 2003
Human CD45^+^ umbilical cord blood cells	CHI	Mouse	1 and 8 days	NSS 1 and 8 days LV 7 days Increased NGF, VEGF expression 35 days	Putative stem cells	Arien-Zakay et al., 2014
Hyperbaric oxygen	WD	Mouse	3 h, 7 days	Astrocyte activation 3 h, 7 days Navigation 7 days NORT 7 days White matter protection 7 days Neuroprotection 7 days	Increased oxygen tension	Baratz-Goldstein et al., 2017
Methamphetamine	LFP	Rats	3.5, 8, and 12 h	White matter 8 h Neuroprotection 12 h Neurogenesis 12 h Motor function 12 h NSS 12 h Navigation 12 h	Increased release of dopamine and other biogenic amines. Inhibition of neuronal vesicular monoamine transporters and monoamine oxidase	Rau et al., 2012, 2014
Minocycline	CCI	Rat	1, 6, 12, and 24 h	Active place avoidance 1 h Navigation 24 h White matter 12 h	Anti-inflammatory, anti-apoptotic, PARP inhibitor	Abdel Baki et al., 2010; Sangobowale M.A. et al., 2018
Minocycline	CHI	Mouse	12 and 24 h	Active place avoidance 12 h Myelin 12 h Induction of oligodendrocyte differentiation 12 h Navigation 24 h	Anti-inflammatory, anti-apoptotic	Sangobowale M. et al., 2018; Sangobowale M.A. et al., 2018
Minocycline N-acetylcysteine	CCI	Rat	1, 6, 12, and 24 h	White matter 6 h Active place avoidance 12 h Navigation 24 h	Anti-inflammatory, anti-apoptotic Anti-oxidant	Abdel Baki et al., 2010; Sangobowale M.A. et al., 2018
Minocycline N-acetylcysteine	CHI	Mouse	12 and 24 h	Active place avoidance acquisition, but not retention 12 h White matter 12 h MAP2 12 h Navigation 24 h	Anti-inflammatory, anti-apoptotic Anti-oxidant	Sangobowale M. et al., 2018; Sangobowale M.A. et al., 2018
MgSO_4_	WD	Rat	0.5, 8, 12, and 24 h	Motor function 24 h	Inhibition of NMDA receptors Downregulation of aquaporin-4 channels	Heath and Vink, 1999
N-acetylcysteine	CHI	Mouse	1 and 12 h	Active place avoidance 12 h	Anti-oxidant	Sangobowale M.A. et al., 2018
N-acetylcysteine	CCI	Rat	1, 6, 12, and 24 h	Navigation 24 h	Anti-oxidant	Sangobowale M.A. et al., 2018
Nicotinamide	CCI	Rats	0.25, 4, 8, and 24 h	LV 4 h Navigation 4 h Astrocyte activation 4 h Motor function 24 h	Vitamin	Hoane et al., 2008a,b; Peterson et al., 2012
NO-1001	CCI	Rat	20–24 h	Microglial Activation Astrocyte activation Motor function	PARP inhibitor	d’Avila et al., 2012
PJ34	CCI	Mouse	3 and 24 h	LV 3 h Motor function 24 h Neuroprotection 24 h Microglial activation 24 h	PARP inhibitor	Stoica et al., 2014
Thymosin β4	CCI	Rat	6 and 24 h	LV 6 h Neurogenesis 6 and 24 h Navigation 24 h Motor Function 24 h NSS 24 h Neuroprotection 24 h White matter 24 h	Sequester actin monomers, tissue repair	Xiong et al., 2012
U-83836e	CCI	Mouse	1, 3, 6, 9, and 18 h	Calpain activation 18 h	Prevention of lipid peroxidation	Mustafa et al., 2011
V-10,367	Impact Acceleration head-injury	Mouse	24 h	Calpain activation Neurodegeneration	Immunophilin ligand	Kupina et al., 2002
Veliparib	CCI	Rat	2 and 24 h	Motor function 24 h Microglial activation 24 h Astrocyte activation 24 h Lower proinflammatory cytokines 24 h Neuroprotection 24 h	Poly (ADP-ribose) polymerase inhibitor	Irvine et al., 2017
Veliparib	CCI	Pig	24 h	Microglial activation	Poly(ADP-ribose) polymerase inhibitor	Irvine et al., 2017

*For each compound, the injury model and species used to test the time to a first dose of the drug after experimental TBI is provided. The therapeutic windows tested are shown with the longest window that improved a given to outcome measure. If known, the mechanism of action for each drug is provided. Abbreviations of injury models are: CCI, controlled cortical impact; CHI, closed head injury; WD, weight drop; LFP, lateral fluid percussion; FPI, fluid percussion injury. Abbreviations of therapeutic outcome measures are: LV, lesion volume; motor outcomes (rotarod, balance beam, posture reflex, grip score, sticky tape, forelimb placing, foot faults, vermicelli test); spatial navigation (Morris water maze, Barnes maze, Y maze); NSS, neuronal severity score; NORT, novel object recognition test, working memory (radial arm maze). Outcome measures are included only if the drug showed an improvement*.

A variety of TBI models have also been developed using ferrets, rabbits, pigs or non-human primates (Dai et al., 2018). Although there are fewer studies using these animals than rodents, they show both rapid changes (apnea, hypertension, elevated intracranial pressure, intracranial bleeds) that is followed by a more delayed gray and white matter injury that is accompanied by a long-lasting inflammatory response (Dai et al., 2018; Vink, 2018). A major advantage of TBI models using larger animals is that they have gyrencephalic brains that better model the mechanical forces that injure the brain after TBI in humans. Thus, large animal studies better model the early pathophysiology of TBI than rodents. The study of TBI in large animals, however, is far more expensive than with rodents and often lack the spectrum of behavioral testing available in rodents. Studies of therapeutic time window frequently need large numbers of animals to achieve statistical significance which may make the use of large animal TBI models prohibitively expensive. The ability of non-rodent models to better model clinical TBI suggests a role to further confirm the therapeutic windows first determined in rodent models.

## Therapeutic Time Window and Loss of Drug Efficacy After TBI

Most drugs lose efficacy with increasing intervals between injury and the time to first treatment. The time course of this decrease in efficacy is known for relatively few drugs (Table 1 and Supplementary Table 1). Most drugs that have been tested in animal TBI models have short therapeutic time windows. The N-type Ca^+2^ channel blocker ziconotide improved mitochondrial function when rats were first dosed 15 min after moderate CCI, but rapidly lost efficacy when first dosed between 1 and 10 h post-injury. These data are consistent with a rapid entry of calcium entry through N-type channels soon after injury (Weber, 2012). Mild hypothermia targets multiple biological processes that makes it an attractive therapy for TBI. Hypothermia inhibits apoptosis, reduces metabolic demand, promotes recovery of energy homeostasis, prevents oxidative damage, maintains the integrity of the blood brain barrier, reduces inflammation, and prevents blood coagulation and seizures. Zhao et al. systematically examined the efficacy of 4 h of hypothermia (32–34°C) in rats beginning 15 min, 2 or 4 h after a moderate CCI (Zhao et al., 2017). Inducing hypothermia within 15 min post-injury produced the largest improvements in behavior (Neurological severity score, Morris water maze), histology (neuroprotection, edema, and neurogenesis) and inflammation (astrocyte activation and neutrophil infiltration). These outcomes also improved when hypothermia was initiated at either 2 or 4 h post-injury, but to a lesser extent than initiating therapy at 15 min. Later time points were not analyzed. Dosing between 3 and 12 h post-injury of methamphetamine has been tested using multiple outcome measures. Multiple behavioral outcomes (Neurological severity score, foot faults, and Morris water maze) were improved with a first dose of methamphetamine at 3 or 7.5 h dosing; 12 h dosing was less effective. As with hypothermia, later time points were not analyzed.

## Therapeutic Time Windows Differ Depending Upon the Animal TBI Model and on the Outcome Measure

Open head models of TBI include controlled cortical impact and fluid percussion injury. These models damage the brain through a craniotomy by directly striking or deforming the dura. The head is immobilized and unable to move (Johnson et al., 2015). Open head models produce a more focal contusion with much less diffuse white matter injury. Closed head models injure the brain by directly striking the head or the skull. In most of these models, the impact produces rapid head movements. Permitting head movement more closely models clinical TBI, and produces a more variable injury than models that prevent head movement (Johnson et al., 2015; Grin’kina et al., 2016). Closed head models produce a smaller focal contusion and a more diffuse white matter injury than open head models. Therapeutic time window has been largely studied using the open head models. Many animal models induce brain injury by selective activation of one element of secondary injury of TBI (i.e., excitotoxicity or oxidative stress). The relevance of therapeutic time window studies in these models to animal models of TBI is unknown. Therefore, only studies of therapeutic time window using animal models of TBI have been discussed in this review.

The therapeutic time window of few drugs have been tested in multiple animal models of TBI. Of the many animal studies showing the efficacy of minocycline (MINO) to improve therapeutic outcomes in experimental TBI, only two dosed MINO using a therapeutic time window of more than 1 h (Garrido-Mesa et al., 2013). A first dose of MINO 4 h after mild injury in a mouse blast model improved acquisition of Morris water maze and decreased levels of multiple biomarkers for glial and neuronal injury in both plasma and in brain tissue (Kovesdi et al., 2012). Later time points were not analyzed, so the therapeutic window of MINO in this blast model has not been fully characterized. In contrast, Sangobowale et al. analyzed the therapeutic time window of MINO in two models, a rat open head controlled cortical impact (CCI) model and a mouse model of closed head injury (CHI). These two models produce overlapping, yet different brain injuries (Johnson et al., 2015). When first dosed with MINO 24 h post-injury, injured rats and mice acquired Barnes maze, a task of spatial navigation (Sangobowale M.A. et al., 2018). Mice, however, did not acquire Barnes maze when MINO was first dosed 72 h after CHI (Whitney, Nikulina, and Bergold, unpublished results). It is unknown if rats could acquire Barnes maze when first dosed at 72 h post-injury. These data suggest that, using Barnes maze as an outcome measure, MINO has a similar therapeutic time window in multiple species and injury models.

Sangobowale M.A. et al. (2018) also tested MINO-treated rats and mice on a second task, active place avoidance. In active place avoidance, rodents avoid a shock zone by attending to distal visual cues while ignoring proximal olfactory cures (Abdel Baki et al., 2010). Active place avoidance could be acquired when MINO was first dosed to mice 12 h after CHI or to rats first dosed 6 h after CCI. Both CCI and CHI induce demyelination of midline commissural tracts. Demyelination of midline commissural tracts prevents acquisition of the active place avoidance (Grin’kina et al., 2013). The therapeutic window for MINO was similar for acquisition of active place avoidance and the prevention of demyelination of midline commissural tracts (Sangobowale M.A. et al., 2018). Thus, outcome measures that have related pathophysiology have similar therapeutic windows. In both models, the therapeutic time window for active place avoidance is shorter than for Barnes maze since active place avoidance requires the coordinated activity of more brain regions than Barnes maze (Cimadevilla et al., 2001; Klur et al., 2009; Shinohara et al., 2012; Grin’kina et al., 2013). This suggests that therapeutic time windows differ depending upon outcome measure. Tasks with higher cognitive demand, such as active place avoidance will have a shorter therapeutic window than simpler tasks, such as Barnes maze. Studies of therapeutic time window should include multiple outcome measures. Given the known heterogeneity of clinical TBI, these observations suggest that the therapeutic window of drugs should be examined using multiple outcome measures in at least two TBI models.

## Combining Drugs Can Extend the Therapeutic Time Window Beyond the Window of Single Drugs

Drug combinations can engage more targets than single drugs, and the ability to engage more targets may lengthen therapeutic time window (Margulies et al., 2016). Minocycline, N-acetylcysteine (NAC) and the minocycline plus N-acetylcysteine (MINO plus NAC) combination were examined in a rat CCI model and a mouse closed head injury model (Table 1). In the rat CCI model, the drug combination of minocycline and N-acetylcysteine was shown to have a longer therapeutic window than the individual drugs (Sangobowale M.A. et al., 2018). Rats acquired active place avoidance if first dosed with MINO at 1 h, but not at 6 h after injury. N-acetylcysteine-treated rats were unable to acquire active place avoidance whether first dosed at 1 or 6 h. Rats treated with MINO plus NAC acquired active place avoidance 12 h post-injury but not at 24 h post-injury. Similar time windows were obtained when retention of myelin was used as an outcome measure (Sangobowale M.A. et al., 2018). In the rat CCI model, the MINO plus NAC drug combination has a longer therapeutic time window than the individual drugs.

## Drugs With Long Therapeutic Windows Reveal Enzymatic and Cellular Processes That Can Be Targeted Hours to Days After TBI

An important, yet understudied question about TBI is what brain functions can be targeted with drugs many hours to days after a TBI. This question can be answered, in part, by analyzing the mode of action of drugs with long therapeutic windows. Multiple drugs with long therapeutic windows target mitochondria, including cyclosporine, FCCP (p-trifluoromethoxyphenylhydrazone), and N-acetylcysteine (Pandya et al., 2009; Sullivan et al., 2011; Sangobowale M.A. et al., 2018). These drugs protect mitochondria in diverse ways; cyclosporine A prevents opening of the mitochondrial permeability transition pore and FCCP uncouples oxidative phosphorylation (Pandya et al., 2009; Sullivan et al., 2011). Both of these drugs lead to inhibition of Ca^+2^ entry into mitochondria. N-acetylcysteine scavenges reactive oxygen species and prevents mitochondrial oxidative stress (Abdel Baki et al., 2010). All three drugs improved outcome measures when first dosed 24 h after injury. These data suggest that drugs that protect mitochondria have long therapeutic windows.

Multiple drugs that inhibit poly (ADP-ribose) polymerase (PARP) also have long therapeutic windows (d’Avila et al., 2012; Stoica et al., 2014; Sangobowale M.A. et al., 2018). In TBI models, PARP is rapidly activated within 30 min after injury yet remains active for many days (LaPlaca et al., 1999). Multiple PARP inhibitors (PJ34, NO-1001, Veliparib, and minocycline) also block microglial activation when dosed up to 24 h post-injury (Table 1). This is due to necessity of PARP activation for microglial activation (d’Avila et al., 2012; Stoica et al., 2014). The effect of Veliparib was observed in both rat and pig CCI models (Irvine et al., 2017). PARP activity is also needed for astrocyte activation and PJ34 and Velinarib prevented astrocyte activation in both rats and pigs (Phulwani and Kielian, 2008; Irvine et al., 2017). PJ34 and NO-1001 improved sensory motor function, but neither PJ34 nor Veliparib improved cognitive outcomes.

Low dose methamphetamine has also shown a broad therapeutic window after TBI. Multiple behavioral and histological outcomes were improved with a first dose of methamphetamine at 3 or 7.5 h dosing. Twelve-hour dosing was less effective than earlier dosing yet the retention of efficacy at 12 h suggests that even later dosing should be tested (Rau et al., 2012, 2014). Methamphetamine acts on multiple vesicular and cell surface transporters to alter neurotransmission of dopamine, norepinephrine, serotonin, and glutamate (Rau et al., 2016). These data suggest that drugs that target multiple neurotransmitter systems may have similarly favorable therapeutic windows.

Chronic neuroinflammation following TBI may also be an effective target for drugs. Byrne et al. showed that mice had chronic microglial activation months after CCI (Byrne et al., 2012). Treatment with the mGluR5 agonist, (RS)-2-chloro-5-hydroxyphenylglycine, beginning 1 month after CCI decreased this neuroinflammation and improved multiple histological and behavioral outcomes. These effects could be blocked by co-administration of a mGluR5 antagonist, providing strong evidence that these effects were through mGluR5 activation.

## Recommendations to Include Therapeutic Window Testing in Preclinical TBI Drug Studies

Pharmacology, safety and potency are key features of any drug to treat TBI (Diaz-Arrastia et al., 2014). A favorable therapeutic time window is also required. Therefore, a careful assessment of therapeutic time window should be performed prior to clinical testing of a drug to treat TBI. We make the following suggestions of how to incorporate testing of therapeutic time window in TBI drug development:

(1) Preclinical drug testing should examine drug efficacy with dosing beginning at progressively longer times after injury. This will analyze whether a drop-off in efficacy is steep or gradual as time to first dose increases.
(2) Preclinical studies of therapeutic time window should include multiple histological, physiological and behavioral outcomes. Drugs are likely to have different therapeutic windows depending upon the outcome measure.
(3) Despite the demonstration that severe or moderate TBI can be dosed as early as 4 h post-injury, drugs with therapeutic windows that are substantially longer than 4 h will be needed to treat the greatest number of brain injury patients.
(4) Patients with mild TBI often delay treatment, therefore drugs to treat mild TBI will require even longer therapeutic windows than drugs to treat moderate to severe TBI.

## Author Contributions

PB conceived and designed the study. MM and PB generated and organized the database. All authors wrote and revised the manuscript and approved the submitted version.

## Conflict of Interest Statement

The authors declare that the research was conducted in the absence of any commercial or financial relationships that could be construed as a potential conflict of interest.
