# Supplementary material for: The Importance of Therapeutic Time Window in the Treatment of Traumatic Brain Injury
Source: Front Neurosci. 2019 Jan 23;13:07. doi: 10.3389/fnins.2019.00007 (PMC6351484; doi:10.3389/fnins.2019.00007)
Supplement: Supplementary file 1 [file Table_1.DOCX]

| Compound | TBI model | Species | Windows tested | Improved outcome measures | Mechanism of action | Reference |
| --- | --- | --- | --- | --- | --- | --- |
| 1400-W | FPI | Rat | 6h | Edema  NSS | iNOS inhibitor | (Louin et al., 2006) |
| 3’,6’-dithiothalidomide | WD | Mouse | 1, 12, 18h | Novelty 12h  Neuroprotection 1h,  Astrogliosis 1h | Inhibition of TNFα | (Baratz et al., 2015) |
| APP96-110 | WD | Rat | 0.5, 5 h | Motor function 5h  White matter 3h  Astrogliosis, 0.5  Microglia 0.5 | Amyloid precursor protein peptide | (Plummer et al., 2018) |
| Atomoxetine | LFP | Rat | 1, 11d | Spatial Navigation 1d | Norepinephrine transporter inhibitor | (Reid and Hamm, 2008) |
| Candesartan | CCI | Mouse | 6h | CBF  Microglia and Neutrophils  Protein Nitration  Neuroprotection  Spatial Navigation | Angiotensin II receptor antagonist | (Villapol et al., 2015) |
| Candesartan | CCI | Mouse | 0.5, 1, 2, 4 hr | NSS 4h | Inhibiting angiotensin II receptor  type 1 | (Timaru-Kast et al., 2012) |
| Carnosic acid | CCI | Mouse | 0.083, 1, 4, or 8 h | Calpain inhibition 8 h | Nrf2–ARE activator | (Miller et al., 2015) |
| 5,5-dimethyl-3(3-fluorophenyl)-4(4-methylsulfonyl) phenyl-2(5H)-furanone | CCI | Rat | 2, 6h | NSS 6h  Spatial Navigation 6h | COX2-specific inhibitor | (Gopez, 2005) |
| Edaravone | CCI | Mouse | 3, 6h | LV 6h,  Lowered nitrotyrosine 6h | Free radical scavenger | (Miyamoto et al., 2013) |
| Erythropoetin | CCI | Rats | 0.5, 6h | Mitochondrial function 6h | Trophic and growth factor | (Xiong et al., 2009) |
| Flavopiridol | LFP | Rat | 0.5, 4h | LV 4h | CDK inhibitor | (Cernak, 2005) |
| Fluasterone | CCI | Rat | 0.5, 2, 12h | Motor function 12h  Spatial navigation12h | Neurosteroid | (Malik et al., 2003) |
| HU-211 | CHI | Rat | 4, 6h | NSS 6h  Motor function 4h  TNFα 4h | Non-competitive NMDA receptor antagonist | (Shohami et al., 1995) |
| Hypothermia | CCI | Rats | 0.25, 2, 4h | NSS: 4h  Spatial navigation 4h  Neutrophils 4h  Neuroprotection 4h  Edema 4h  Neurogenesis 4h  Astrogliosis 4h  BCL2/BAX ratio 4h  Neurogenesis 4h  Caspases 4h | Reduction of intracranial pressure, increased cerebral metabolism and perfusion, decreased excitatoxicity and pro-inflammatory cytokines | (Zhao et al., 2017) |
| L-argininase | CCI | Rat | 5min, 1, 6, 24, 48h | LV 1h  Neuroprotection 5 min | Increasing cerebral blood flow | (Cherian et al., 2003) |
| L-NIL | FPI | Rat | 6h | Edema  NSS | iNOS inhibitor | (Louin et al., 2006) |
| Lithium | CCI | Mouse | 3, 6h | LV 3h | Modulation of neurotransmission and inositol signaling, prevents oxidative stress | (Yu et al., 2012) |
| MDL-28170 | MFP | Rat | 0.5, 2, 4h | White matter 0.5h  β-APP staining 4h | Calpain II inhibitor | (Ai et al., 2007) |
| MDL-28170 | CCI | Mouse | 0.25, 1, 3h | Calpain activation 1h | Calpain II inhibitor | (Thompson, 2010) |
| Melanocortins | Impact-Accelleration | Rats | 3h, 6h | Lowered pro-apopotic proteins 6h  Increased anti-apoptotic proteins 6h  Anti-inflammatory cytokines 6h  Neuroprotection 6h  White matter 6h  Spatial Navigation 6h  Sensory motor orientation 3h, 6h | Peptide hormone | (Bitto et al., 2012) |
| MW151 | CHI | Mouse | 3h, 6h | IL-1β 6h  Working memory 3h  Astrogliosis 3h  Microglia 3h | Prevents pro-inflammatory cytokine expression | (Bachstetter et al., 2015) |
| Nicotinamide plus progesterone | CCI | Rat | 4h | Motor function  Astrogliosis  Neuroprotection | Vitamin plus neurosteroid | (Peterson et al., 2015) |
| Progesterone | CCI | Rat | 4h | Motor function  LV  Neuroprotection  Astrogliosis | Neurosteroid | (Peterson et al., 2012;Peterson et al., 2015) |
| Progesterone | WD | Rat | 6h | Decreased GAP-43 and Nogo expression  Astrogliosis | Neurosteroid | (Liu et al., 2014) |
| Pycnogenol | CCI | Rat | 0.25, 2, 4h | Lipid peroxidation, 4h  Synapse protein expression 4h | Anti-oxidant | (Ansari, 2013) |
| Resatorvid | WD | Mouse | 4h | Motor function  Neuroprotection | Toll-Like Receptor 4 inhibitor | (Zhang, 2014) |
| Riluzole | FPI | Rat | 6h | Edema | Na^+^ channel inhibitor | (Wahl and Stutzmann, 1999) |
| SJA6017 | WD | Mouse | 4, 6h | Motor function 4h | Calpain I and II Inhibitor | (Kupina et al., 2001) |
| SNJ-1945 | CCI | Mouse | 0.25, 1h, 2h | Calpain Inhibition 0.25h | Calpain inhibitor | (Bains et al., 2013) |
| Telmisartan | CCI | Mouse | 6h | Cerebral Blood Flow  Microglia and Neutrophils  Protein Nitration  Neuroprotection | Angiotensin II receptor antagonist | {Villapol, 2015 #10. |
| Xenon | CCI | Mouse | 0.25, 1, 3, 6h | NSS 1h  LV 3h | NMDA receptor antagonist | (Campos-Pires et al., 2015) |
| z-DEVD-fmk | CCI | Mouse | 1, 4, 8, 24h | LV 1h  Spatial navigation 1h  Motor function 1h | Caspase inhibitor | (Knoblach et al., 2004) |
| Ziconotide | CCI | Rat | 0.25, 1, 2, 4, 6, 10h | Mitochondrial function 0.25h | N-type calcium channel blocker | (Verweij et al., 2000) |

**Supplementary Table** **Drugs with a therapeutic time window of between 4 hours and 12 hours greater in animal models of TBI**. For each compound, the time after injury to a first dose of the drug to test for therapeutic time window is provided. Shown are injury model and species used. The therapeutic windows tested are shown along with the longest window that improved a given to outcome measure. The mechanism of action for each drug is provided, if known. Injury model abbreviations: CCI-Controlled Cortical Impact; CHI-Closed Head Injury; WD- Weight Drop; LFP- Lateral Fluid Percussion; FPI- Fluid Percussion Injury. Therapeutic outcome measure abbreviations: LV- Lesion volume; Motor outcomes (Rotarod, Balance Beam, Posture Reflex, Grip score, Sticky tape, Forelimb placing, Foot faults, Vermicelli test); Spatial Navigation (Morris water maze, Barnes maze, Y maze) NSS-Neuronal severity score, NORT-Novel object recognition test, working memory (radial arm maze). Only outcome measures that indicated a therapeutic improvement are included.

Literature Cited in Supplementary Table

Ai, J., Liu, E., Wang, J., Chen, Y., Yu, J., and Baker, A.J. (2007). Calpain inhibitor MDL-28170 reduces the functional and structural deterioration of corpus callosum following fluid percussion injury. *J Neurotrauma* 24**,** 960-978.

Ansari, M.A., Roberts, K.N., Scheff, S.W. (2013). Dose- and Time-Dependent Neuroprotective Effects of Pycnogenol(®) following Traumatic Brain Injury. *J. Neurotrauma* 30**,** 1542-1549.

Bachstetter, A.D., Webster, S.J., Goulding, D.S., Morton, J.E., Watterson, D.M., Van Eldik, L.J. (2015). Attenuation of traumatic brain injury-induced cognitive impairment in mice by targeting increased cytokine levels with a small molecule experimental therapeutic. *J. Neuroinflam* 12**,** 69.

Bains, M., Cebak, J.E., Gilmer, L.K., Barnes, C.C., Thompson, S.N., Geddes, J.W., Hall, E.D. (2013). Pharmacological analysis of the cortical neuronal cytoskeletal protective efficacy of the calpain inhibitor SNJ-1945 in a mouse traumatic brain injury model. *J Neurochem* 125**,** 125-132.

Baratz, R., Tweedie, D., Wang, J.Y., Rubovitch, V., Luo, W., Hoffer, B.J., Greig, N.H., Pick, C.G. (2015). Transiently lowering tumor necrosis factor-alpha synthesis ameliorates neuronal cell loss and cognitive impairments induced by minimal traumatic brain injury in mice. *J Neuroinflam* 12**,** 45.

Bitto, A., Polito, F., Irrera, N., Calo, M., Spaccapelo, L., Marini, H.R., Giuliani, D., Ottani, A., Rinaldi, M., Minutoli, L., Guarini, S., Squadrito, F., Altavilla, D. (2012). Protective effects of melanocortins on short-term changes in a rat model of traumatic brain injury. *Crit Care Med* 40**,** 945-951.

Campos-Pires, R., Armstrong, S.P., Sebastiani, A., Luh, C., Gruss, M., Radyushkin, K., Hirnet, T., Werner, C., Engelhard, K., Franks, N.P., Thal, S.C., Dickinson, R. (2015). Xenon improves neurologic outcome and reduces secondary injury following trauma in an in vivo model of traumatic brain injury. *Crit Care Med* 43**,** 149-158.

Cernak, I., Stoica, B., Byrnes, K.R., Di Giovanni, S., Faden, A.I. (2005). Role of the cell cycle in the pathobiology of central nervous system trauma. *Cell Cycle* 4**,** 1286-1293.

Cherian, L., Chacko, G., Goodman, C., Robertson, C.S. (2003). Neuroprotective effects of L-arginine administration after cortical impact injury in rats: dose response and time window. *J Pharmacol Exp Ther* 304**,** 617-623.

Gopez, J.J., Yue, H., Vasudevan, R., Malik, A.M., Fogelsanger, L.N., Lewis, S., Panikashvili, D., Shohami, E., Jansen, S.A., Narayan, R.K., Strauss, K.I. (2005). Cyclooxygenase-2-specific Inhibitor Improves Functional Outcomes, Provides Neuroprotection, and Reduces Inflammation in a Rat Model of Traumatic Brain Injury. *Neurosurgery* 56**,** 590–604.

Knoblach, S.M., Alroy, D.A., Nikolaeva, M., Cernak, I., Stoica, B.A., Faden, A.I. (2004). Caspase inhibitor z-DEVD-fmk attenuates calpain and necrotic cell death in vitro and after traumatic brain injury. *J Cereb Blood Flow Metab* 24**,** 1119-1132.

Kupina, N.C., Nath, R., Bernath, E.E., Inoue, J., Mitsuyoshi, A., Yuen, P.W., Wang, K.K., Hall, E.D. (2001). The novel calpain inhibitor SJA6017 improves functional outcome after delayed administration in a mouse model of diffuse brain injury. *J Neurotrauma* 18**,** 1229-1240.

Liu, F., Liao, F., Li, W., Han, Y., Liao, D. (2014). Progesterone alters Nogo-A, GFAP and GAP-43 expression in a rat model of traumatic brain injury. *Mol Med Rep* 9**,** 1225-1231.

Louin, G., Marchand-Verrecchia, C., Palmier, B., Plotkine, M., Jafarian-Tehrani, M. (2006). Selective inhibition of inducible nitric oxide synthase reduces neurological deficit but not cerebral edema following traumatic brain injury. *Neuropharm* 50**,** 182-190.

Malik, A.S., Narayan, R.K., Wendling, W.W., Cole, R.W., Pashko, L.L., Schwartz, A.G., and Strauss, K.I. (2003). A novel dehydroepiandrosterone analog improves functional recovery in a rat traumatic brain injury model. *J Neurotrauma* 20**,** 463-476.

Miller, D.M., Singh, I.N., Wang, J.A., Hall, E.D. (2015). Nrf2-ARE activator carnosic acid decreases mitochondrial dysfunction, oxidative damage and neuronal cytoskeletal degradation following traumatic brain injury in mice. *Exp Neurol* 264**,** 103-110.

Miyamoto, K., Ohtaki, H., Dohi, K., Tsumuraya, T., Song, D., Kiriyama, K., Satoh, K., Shimizu, A., Aruga, T., and Shioda, S. (2013). Therapeutic Time Window for Edaravone Treatment of Traumatic Brain Injury in Mice. *BioMed Res Int* 2013**,** 13.

Peterson, T.C., Anderson, G.D., Kantor, E.D., Hoane, M.R. (2012). A comparison of the effects of nicotinamide and progesterone on functional recovery of cognitive behavior following cortical contusion injury in the rat. *J Neurotrauma* 29**,** 2823-2830.

Peterson, T.C., Hoane, M.R., Mcconomy, K.S., Farin, F.M., Bammler, T.K., Macdonald, J.W., Kantor, E.D., Anderson, G.D. (2015). A Combination Therapy of Nicotinamide and Progesterone Improves Functional Recovery following Traumatic Brain Injury. *J Neurotrauma* 32**,** 765-779.

Plummer, S.L., Corrigan, F., Thornton, E., Woenig, J.A., Vink, R., Cappai, R., Van Den Heuvel, C. (2018). The amyloid precursor protein derivative, APP96-110, is efficacious following intravenous administration after traumatic brain injury. *PLoS One* 13**,** e0190449.

Reid, W.M., and Hamm, R.J. (2008). Post-injury atomoxetine treatment improves cognition following experimental traumatic brain injury. *J Neurotrauma* 25**,** 248-256.

Shohami, E., Novikov, M., Bass, R. (1995). Long-term effect of HU-211, a novel non-competitive NMDA antagonist, on motor and memory functions after closed head injury in the rat. *Brain Res* 674**,** 55-62.

Thompson, S.N., Carrico, K.M., Mustafa, A.G., Bains, M., Hall, E.D. (2010). A Pharmacological Analysis of the Neuroprotective Efficacy of the Brain- and Cell-Permeable Calpain Inhibitor MDL-28170 in the Mouse Controlled Cortical Impact Traumatic Brain Injury Model. *J. Neurotrauma* 27**,** 2233-2243.

Timaru-Kast, R., Wyschkon, S., Luh, C., Schaible, E.V., Lehmann, F., Merk, P., Werner, C., Engelhard, K., Thal, S.C. (2012). Delayed inhibition of angiotensin II receptor type 1 reduces secondary brain damage and improves functional recovery after experimental brain trauma. *Crit Care Med* 40**,** 935-944.

Verweij, B.H., Muizelaar, J.P., Vinas, F.C., Peterson, P.L., Xiong, Y., Lee, C.P. (2000). Improvement in mitochondrial dysfunction as a new surrogate efficiency measure for preclinical trials: dose-response and time-window profiles for administration of the calcium channel blocker Ziconotide in experimental brain injury. *J Neurosurg* 93**,** 829-834.

Villapol, S., Balarezo, M.G., Affram, K., Saavedra, J.M., Symes, A.J. (2015). Neurorestoration after traumatic brain injury through angiotensin II receptor blockage. *Brain* 138**,** 3299-3315.

Wahl, F., and Stutzmann, J.M. (1999). Neuroprotective effects of riluzole in neurotrauma models: a review. *Acta Neurochir Suppl* 73**,** 103-110.

Xiong, Y., Chopp, M., and Lee, C.-P. (2009). Erythropoietin improves brain mitochondrial function in rats after traumatic brain injury. *Neurol Res* 31**,** 496-502.

Yu, F., Wang, Z., Tchantchou, F., Chiu, C.T., Zhang, Y., Chuang, D.M. (2012). Lithium ameliorates neurodegeneration, suppresses neuroinflammation, and improves behavioral performance in a mouse model of traumatic brain injury. *J Neurotrauma* 29**,** 362-374.

Zhang, D., . Li, H., Li, T., Zhou, M., Hao, S., Yan, H., Yu, Z., Li, W., Li, K., Hang, C. (2014). TLR4 inhibitor resatorvid provides neuroprotection in experimental traumatic brain injury: Implication in the treatment of human brain injury. *Neurochem Int* 75**,** 11-18.

Zhao, W.Y., Chen, S.B., Wang, J.J., Xu, C., Zhao, M.L., Dong, H.J., Liang, H.Q., Li, X.H., Tu, Y., Zhang, S., Chen, C., Sun, H.T. (2017). Establishment of an ideal time window model in hypothermic-targeted temperature management after traumatic brain injury in rats. *Brain Res* 1669**,** 141-149.
